# Supplementary material for: Quality Assessment of Untargeted Analytical Data in a Large-Scale Metabolomic Study
Source: J Clin Med. 2021 Apr 22;10(9):1826. doi: 10.3390/jcm10091826 (PMC8122759; doi:10.3390/jcm10091826)
Supplement: Supplementary file 1 [file jcm-10-01826-s001.zip › jcm-1157315-supplementary.pdf]

Dilution rate designation	(1) MeOH (uL)	(2) Milli-Q (uL)	(3) Sample (uL)
X10	450	0	50
X20	450	25	25
X39	450	37	13
X83	450	44	6
X167	450	47	3

Plasma dilution table

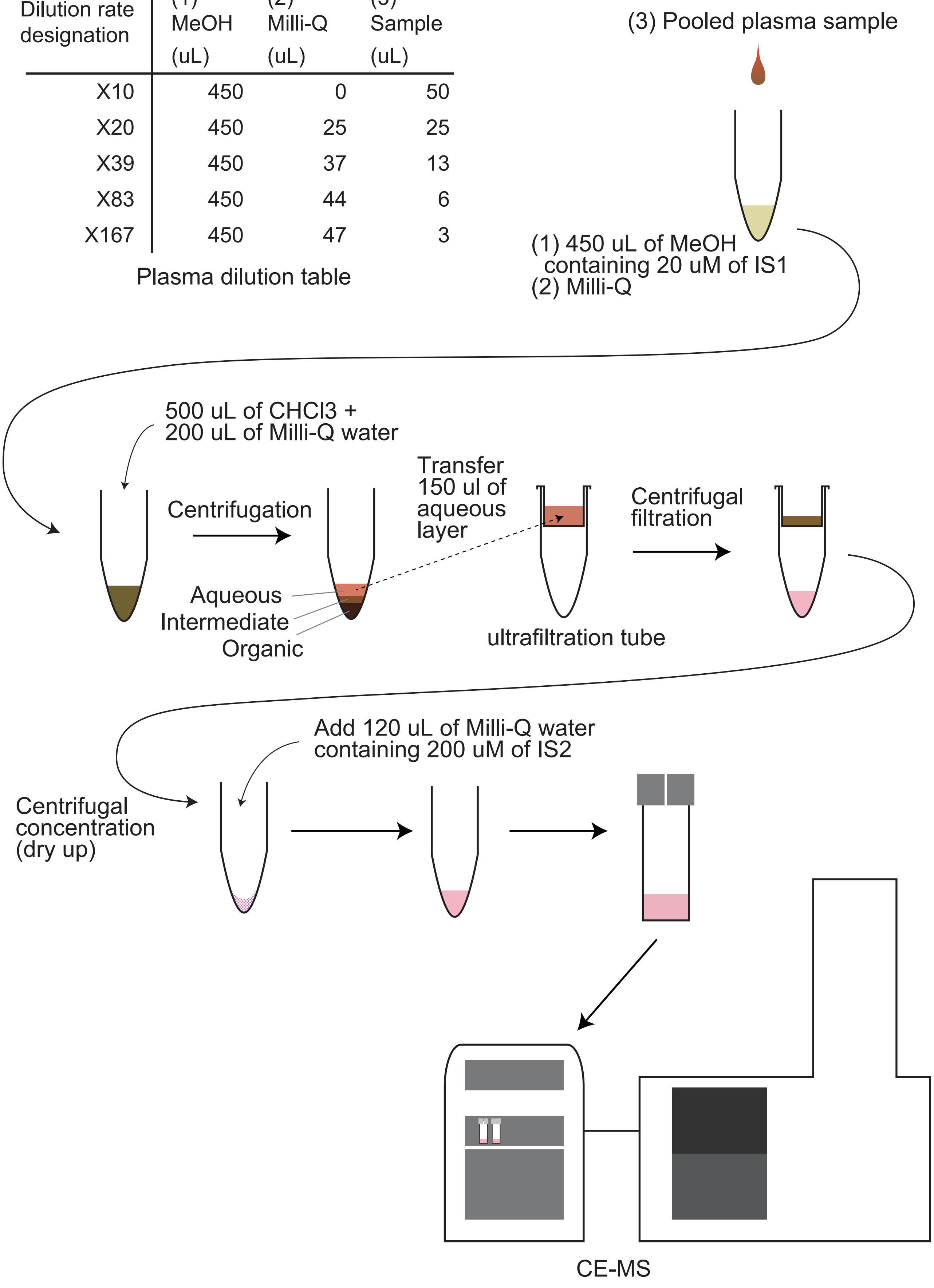

Supplemental Figure S1: Procedure used to dilute pooled plasma samples

Dilution rate designation	(1) Milli-Q (uL)	(2) Milli-Q containing ISs (uL)	(3) Urine (uL)
X5	140	20	40
X10	160	20	20
X20	170	20	10
X40	175	20	5
X100	178	20	2

Urine dilution table

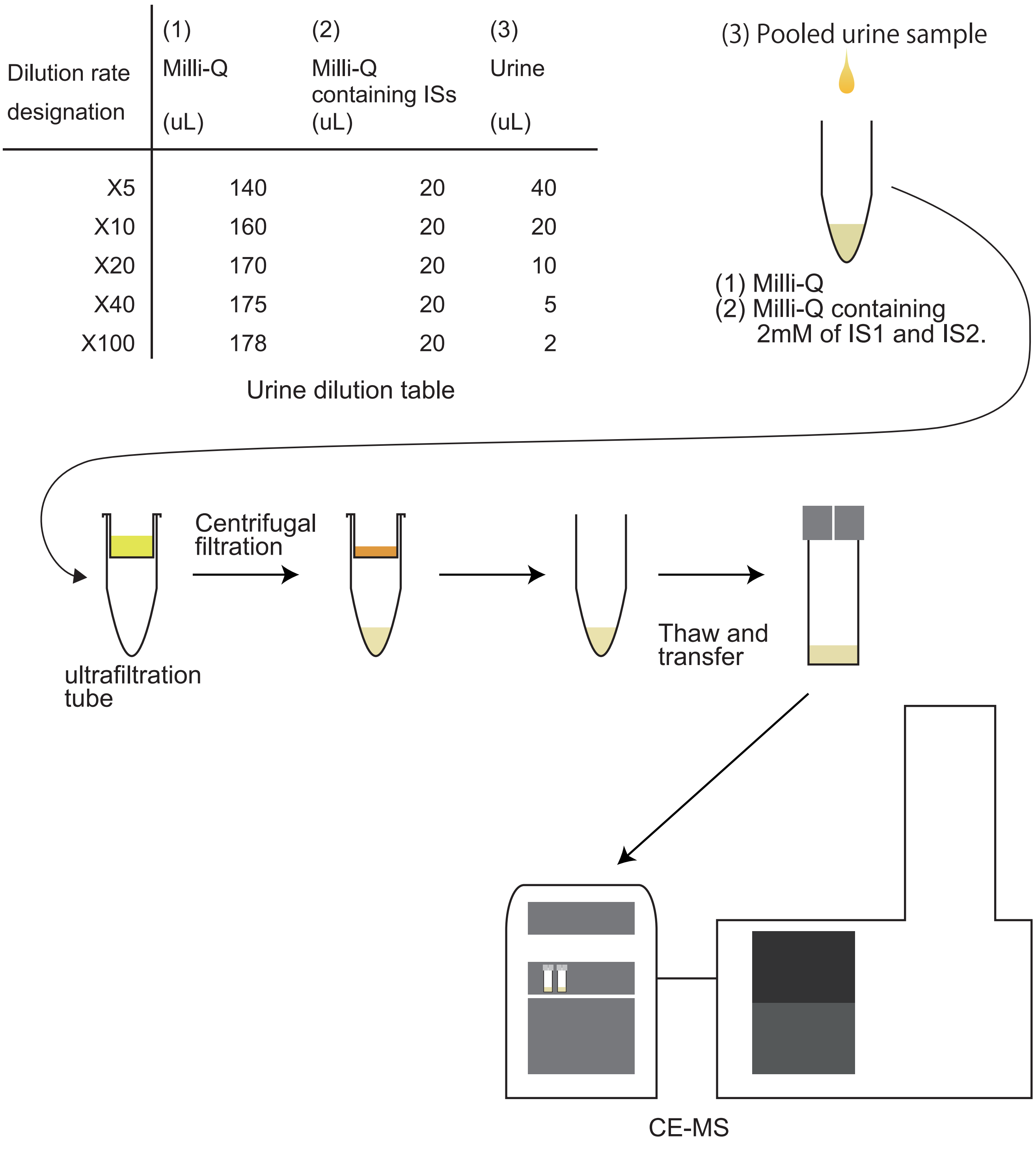

Supplemental Figure S2: Procedure used to dilute of pooled urine samples

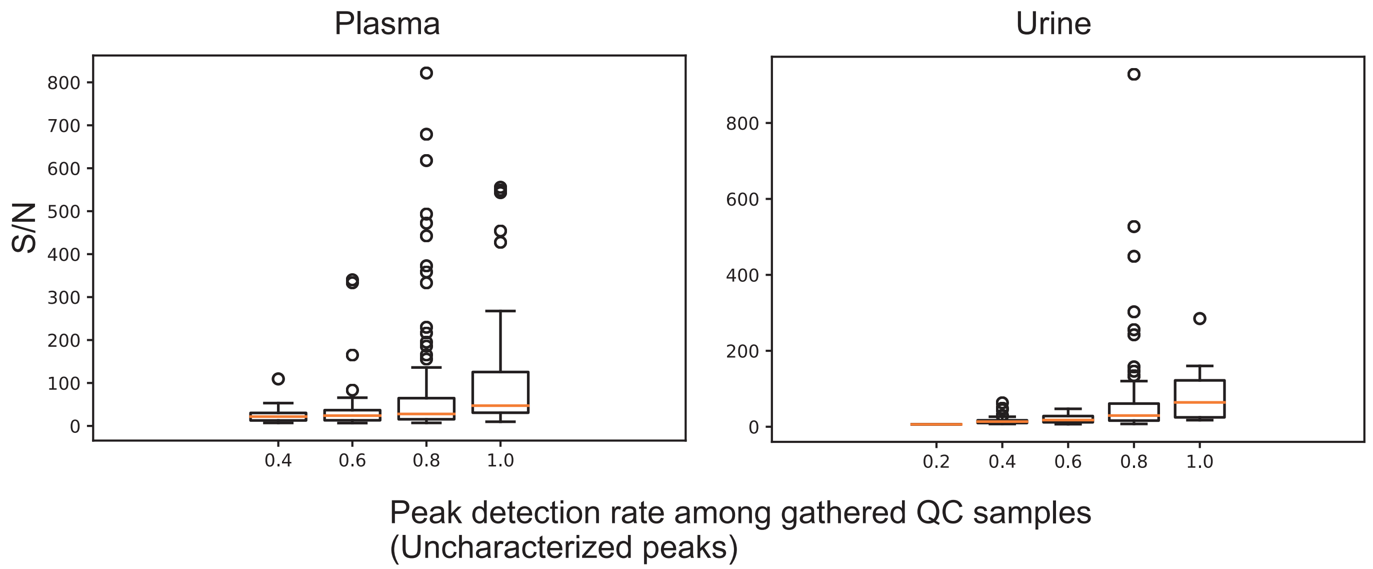

Supplemental Figure S3: Relationship between detection rates of the peaks among the gathered quality control (QC) samples and their signal-to-noise (S/N) ratios

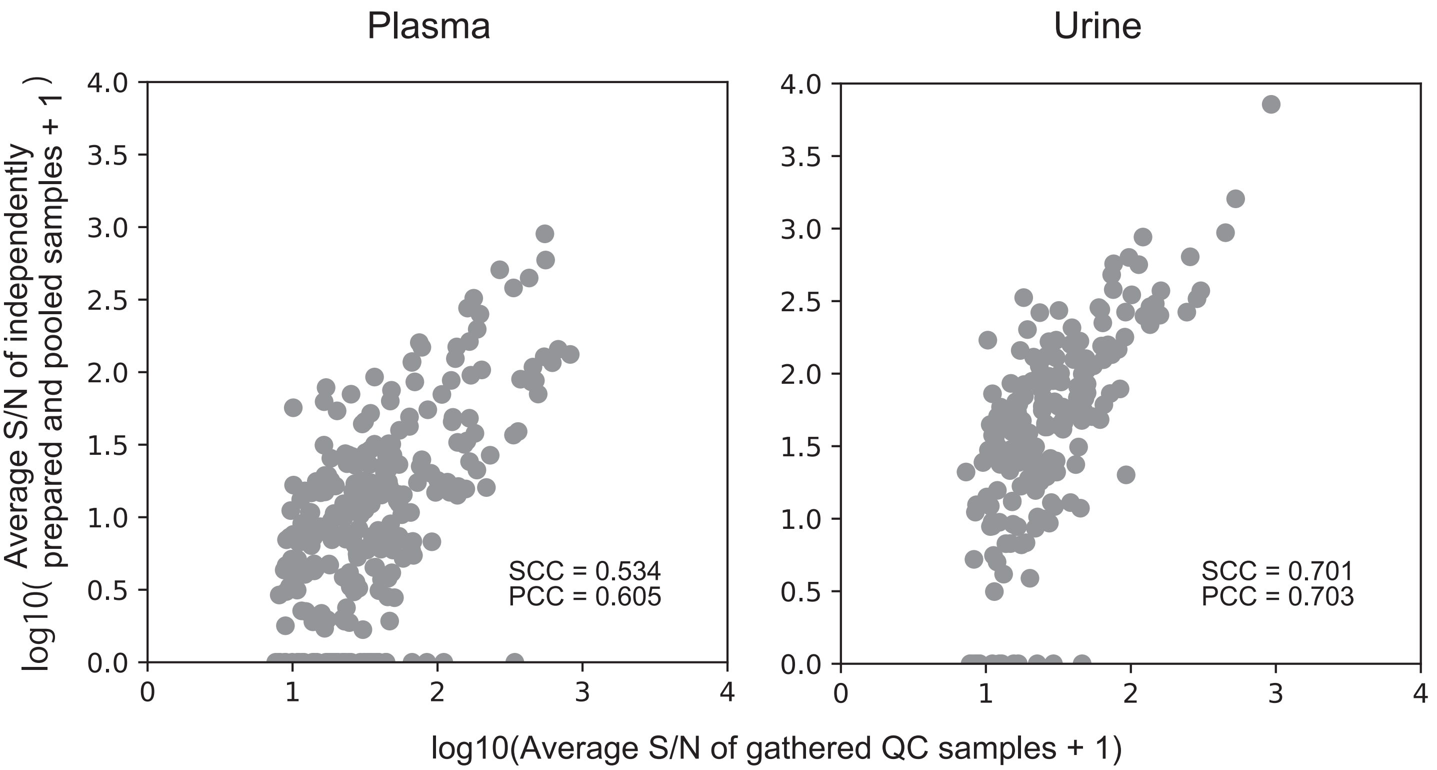

Supplemental Figure S4: Correlation between average signal-to-noise (S/N) ratios of the peaks in the gathered quality control (QC) samples and those in independently prepared and pooled samples
